# Spark Plasma Sintering of Aluminum Nanocomposite Powders: Recent Strategy to Translate from Lab-Scale to Mass Production

**DOI:** 10.3390/nano11123372

**Published:** 2021-12-12

**Authors:** Roberto Hernández-Maya, Nicolás Antonio Ulloa-Castillo, Oscar Martínez-Romero, Emmanuel Segura-Cárdenas, Alex Elías-Zúñiga

**Affiliations:** 1 Siemens, Research and Development Department, Libramiento Arco Vial Poniente Km 4.2, Santa Catarina 66350, Nuevo León, Mexico; roberto.hernandez_maya@siemens.com; 2 Tecnologico de Monterrey, Department of Mechanical Engineering and Advanced Materials, School of Engineering and Sciences, Av. Eugenio Garza Sada Sur 2501, Monterrey 64849, Nuevo León, Mexico; nicolas.ulloa@tec.mx (N.A.U.-C.); esca@tec.mx (E.S.-C.)

**Keywords:** aluminum nanocomposite material, multi-walled carbon nanotubes (MWCNTs), reinforced Al powders, from lab to mass production, scale up process, industrial implementation process

## Abstract

The aim of this paper focuses on presenting a recent study that describes the fundamental steps needed to effectively scale-up from lab to mass production parts produced from Al powders reinforced with 0.5 wt% of industrial multiwalled carbon nanotubes (MWCNTs), with mechanical and electrical conductivity properties higher that those measured at the lab scale. The produced material samples were produced via a Spark Plasma Sintering (SPS) process using nanocomposite aluminum powders elaborated with a planetary ball-mill at the lab scale, and high-volume attrition milling equipment in combination with controlled atmosphere sinter hardening furnace equipment, which were used to consolidate the material at the industrial level. Surprisingly, the electrical conductivity and mechanical properties of the samples produced with the reinforced nanocomposite Al powders were made with mass production equipment and were similar or higher than those samples fabricated using metallic powders prepared with ball-mill lab equipment. Experimental measurements show that the hardness and the electrical conductivity properties of the samples fabricated with the mass production Al powders are 48% and 7.5% higher than those of the produced lab samples. This paper elucidates the steps that one needs to follow during the mass production process of reinforced aluminum powders to improve the physical properties of metallic samples consolidated via the SPS process.

## 1. Introduction

In the few last years, there has been an exhaustive effort to developed advanced composite metallic materials with improved mechanical, electrical, tribology, and thermal properties, that monolithic materials cannot provide per se. Therefore, the addition of two of more materials need to be combined in order to achieve improved properties. In this sense, several studies have proved that aluminum matrix material reinforced with carbon nanotubes (CNTs) can have a better thermal, electrical, and mechanical performance when compared to other metallic materials [1,2,3]. However, one of the main challenges of developing composite materials consists of translating the lab experimental observations of functional prototypes to an industrial large-scale production process considering cost-effective manufacturing technologies [4,5] and solving several difficulties associated with the dispersion, degradation, and interface strength between the Al matrix and the added reinforcements that could reduce the composite physical and thermal properties [6]. In order to overcome these obstacles, several techniques have been developed to avoid CNT degradation and to get homogenous dispersion when added into the Al matrix [2,3,7]. Furthermore, manufacturing processes, such as Spark plasma extrusion (SPE) and Spark Plasma Sintering (SPS), are becoming popular because the pressure variation and applied temperatures consolidate the nanocrystalline materials well and provide an effective interface formation [8,9]. Arunachalam et al. provided a good discussion of the manufacturing process used for the consolidation of metal matrix composites [5] such as spark plasma sintering, high energy ball mill mixing and sintering, vacuum gas sintering, microwave sintering, liquid state processing, infiltration methods, casting methods, semi-solid process, compocasting, rheocasting, spray deposition, and screw extruders, to name a few. Among these methods, Arunachalam and co-workers recommended, based on the results reported by researchers in the literature, a stir-casting furnace design, claiming that this system is capable of a uniform distribution of the added nano reinforcement. However, they did not provide any evidence of having a cost-effective industrial production process. In this sense, Sharma et al. [1] identified that aluminum metal matrix composites are one of the most popular and common metals, with major applications in transportation vehicles, the aerospace industry, defense weapons, electronics and optical instruments because of its physical, thermal, electrical, tribological, and structural properties. They concluded that not only the raw material and its reinforcement costs are important factors in producing metal matrix composites, but also the fabrication process of the product that is expected to be robust and cost-effective.

On the other hand, several attempts have been reported in the literature [10] to move from a lab-scale production of equipment to one for industrial production, since the continuous process of SPS offers great advantages in industrial settings to process metallic powder-based materials to manufacture complex shapes. At present, in Siemens Monterrey, in close collaboration with some providers, the previous results [2,3] were used for the consolidation of aluminum metallic powders reinforced with MWCNTs, in a cost-effective way, with results that are very encouraging, since the properties of the consolidated specimens concur with industrial standards in relation to their physical, thermal and electrical properties.

Therefore, in the present work, we address the main aspects that were considered to translate the process from the laboratory scale to an industrial scale-up environment.

## 2. Materials and Methods

Aluminum powder with purity >99% was purchased from Ampal Inc., Palmerton, PA, USA. Industrial-grade MWCNTs were purchased from Nanostructured and Amorphous Materials, Inc., Houston, TX, USA. The MWCNTs possess outer diameters, which range from 50 to 80 nm, inner diameters from 5 to 15 nm and lengths between 10–20 μm. Stearic acid (CH_3_(CH_2_)_16_COOH) used as a process control agent (PCA) was purchased from Sigma Aldrich, St. Louis, MO, USA. All materials were used without any further purification.

### 2.1. Ball-Milling Powder Preparation and SPS Sintering Consolidation at Lab Scale

The Al-based nanocomposite powders were processed through planetary ball-mill equipment (Retsch, PM 100), purchased from Fisher Scientific (Loughborough, Leicestershire, UK)) and considering a reinforcement of 0.5 wt% of MWCNTs. The ball-milling processing of powder was performed following the methodology previously reported in [3], which consisted of adding 0.5 wt% of MWCNTs in the Al matrix powder and using a ball-to-powder ratio of 10:1. The equipment was operated at 360 rpm at different milling times corresponding to 5, 10, 15 and 20 min, in order to reproduce the morphology previously reported in [3]. The sintering consolidation of Al-based nanocomposite powders was achieved by using a SPS Dr. Sinter model SPS-1050 equipment, (Fuji Electronic Industrial, Tsurugashima, Saitama, Japan). The procedure and sintering conditions implemented are described in [2,3]. In general, the procedure consisted of adding the nanocomposite powder in a graphite die and compacted at 50 MPa with the aid of graphite punches. The sintering was completed when the temperature reached 620 °C (heating rate of 50 °C/min) with a holding time of 5 min and through operating the SPS system. In total, a batch of 10 disk-shaped specimens (M1–M10) of 40 mm in diameter with 10 mm thickness were sintered by SPS. All sintered samples were made with the Al-based nanocomposite powders ball-milled at 360 rpm for 5 min.

### 2.2. Morphology Characterization

An SEM microscope with energy dispersive spectroscopy (EDS) ZEISS model EVO MA 25 (Carl Zeiss Microscopy Ltd, Cambridge, UK) was used to investigate the morphology of Al-based nanocomposite materials. The micrographs were taken considering secondary electrons (SE) and backscattered electrons (BSE), which is relevant to explore the chemical composition in the nanocomposite materials. In this sense, energy dispersive spectroscopy (EDS) elemental mappings were analyzed to investigate the Carbon distribution in the sintered nanocomposites. The corresponding micrographs were collected operating the system at 20 kV as a acceleration voltage.

### 2.3. Densification

The consolidation of sintered nanocomposites was investigated by analyzing their densification. The experimental density (*ρ_e_*) was performed following a methodology based on Archimedes’ principle, using deionized water as the immersion medium and in accordance with the ASTM B962–17 standard norm. The corresponding values were calculated using the following equation:

(1) $$\rho_{e}=\frac{A\;\rho_{w}}{A-\left(B-C\right)},$$

where *A* is the mass of the sample, *B* is the mass of the oil-impregnated sample, *C* is the mass of the oil-impregnated sample immersed in water, and *ρ_w_* is the density of water (0.9956 g/cm^3^). In the same way, the relative density (*ρ_r_*) values were calculated using

(2) $$\rho_{r}=\frac{\rho_{e}}{\rho_{c}}\times 100,$$

where *ρ_c_* is the theoretical density, which can be assessed by considering the rule of mixtures *ρ_c_* = *ρ*_CNT_ *V*_CNT_ + *ρ*_Al_ *V*_Al_, where *ρ*_CNT_ and *ρ*_Al_ are the theoretical carbon nanotubes and aluminum densities, respectively, *V* is the volume fraction of either, MWCNT (2.1 g/cm^3^) or Al (2.7 g/cm^3^) matrix.

### 2.4. Roughness Characterization

The roughness analysis of the sintered specimens was carried out through an Alicona Infinite Focus G4 microscope purchased from Imaging GmbH (Graz, Austria). The images were taken with a 20× lens magnification, which allows a vertical resolution of 50 μm and a lateral resolution of 3.5 μm. The corresponding roughness values were retrieved from the average of 10 measurements in accordance with the ISO 4287 standard norm. In this work, the average roughness (*Ra*) is calculated using a cut-off length (Lc) filter of 80 µm, which is optimal for the vertical and lateral resolution achieved with the 20× lens magnification.

### 2.5. Vickers Hardness Tests

Vickers hardness tests were carried out using a micro-hardness tester model ZHVµ ZwickRoell GmbH and Co. KG (Ulm, Germany). The hardness tests were performed using a diamond indenter with a square-based pyramid base (136° between faces) and by applying a force 50 gf (0.98 N) on the sintered nanocomposite specimens during 10 s. The tests were performed in accordance with the ISO 6507 and ASTM E384 standard norms considering the average of 5 measurements. Such values were used to calculate the tensile and yield strength values, following the methodology described by Cahoon et al. [11] and using the following expressions:

(3) $$T=\left(\frac{H_{m}}{2.9}\right)\times {\left(\frac{n}{0.217}\right)}^{n}\;,$$

(4) $$Y=\left(\frac{H_{m}}{3}\right)\times {\left(0.1\right)}^{n},$$

where *T* is the tensile strength of the material (MPa), *Y* is yield strength of the material (MPa), *H_m_* is microhardness of the material (N/m^2^) and *n* is the strain-hardening coefficient of the material, taken as 0.2 as reported by Callister [12].

### 2.6. Electrical Conductivity

The electrical conductivity characterization was performed using a Sigmascope instrument, model SPM10 purchased from Helmut Fischer GMBH (Sindelfingen, Germany). The measurements were performed in accordance with the ASTM E 1004 and DIN EN 2004-1 norms and considering the average value from three different zones of the nominal cross-section area of obtained sintered nanocomposites.

## 3. Recent Process to Translate Nanocomposite Powders from the Lab Scale to the Industrial Scale: Important Aspects

As previously mentioned, the main challenge of scaling the production of consolidated metal-based nanocomposites is to achieve the desirable mechanical and physical properties to produce reliable parts that fulfill engineering codes, standards, and industry regulations. However, to scale a new developed material from the laboratory to the company production ramp-up is not a straightforward process, since several critical experimental factors need to be taken into account before translating the experimental laboratory knowledge to the scale production, including the adoption of new production technologies or equipment conversion to produce parts with the developed reinforced material.

In the case of the industrial scale developed Aluminum-based nanocomposites, we have followed similar experimental conditions to those previously discussed in Section 2.1 for the homogeneous dispersion of the carbon nanotubes into metallic powders, but instead of using the planetary ball-mill equipment (Retsch, PM 100), now a high material volume in attrition milling equipment (Union Process Inc., Akron, OH, USA) was used. The equipment was operated at 215 rpm, with 5 min as a holding time, and with a load of 2700 gr per batch. To make economically attractive the development of a functional part using the developed material in a lab scale environment, it is necessary to identify existing technologies or to develop new processes to achieve the desirable part properties. Although the milling method, ball and attrition milling processes could have similar results, the attrition milling process has some advantages against the ball milling process. The more important ones include less energy consumption, less holding time, and better distribution on the facility space; these mentioned points are traduced on a less expensive process for powder preparation; a similar comparison was previously reported in [13]. Furthermore, in industrial scale environments, the presence of a process control agent (PCA) for the milling process is needed to improve the tribological properties of the produce powders. Therefore, we have added 0.5 wt% of stearic acid as a PCA to tune the milling method from the lab scale to the industrial scale. This should be done to achieve repeatability during the mass production of the Al-based nanocomposite powders, and for obtaining homogenous dispersion of the CNTs within the metallic matrix as well as for avoiding undesired cold welding processes [14,15]. On the other hand, the Al-based nanocomposite powder sintering consolidation was carried out in 12 in controlled atmosphere sinter hardening furnace equipment (Webster-Hoff Corporation, Glendale Heights, IL, USA). The functional parts were compressed under an uniaxial mode on a mold made from a steel tool, with electropolished surface before the sinter step. The controlled atmosphere furnace can produce a huge batch of material in shorter times, and also allows the extraction of the produced gas from the pieces due to the degradation of the PCA. For functional pieces made from composite materials to substitute common materials, the capabilities to produce larger batches will influence the adaptability of new materials in several engineered fields; the general methodology to produce the industrial scale-up parts is: Milling process → Compaction → Sintering → Secondary Operations. 

## 4. Results and Discussion

### 4.1. Morphological Analysis of Laboratory Ball-Milled Nanocomposite Powder

**Figure 1:**
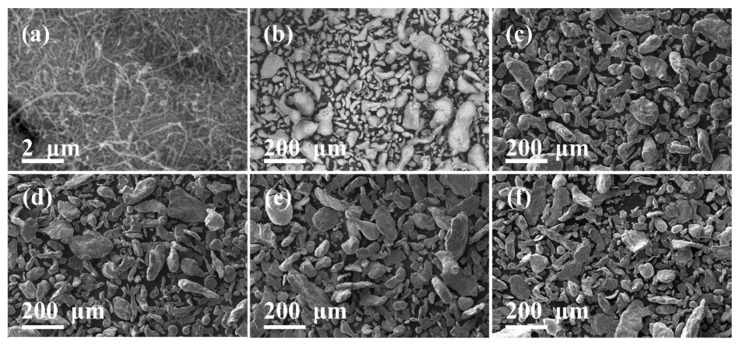
SE-SEM micrographs for as-received: (**a**) MWCNTs and (**b**) Al powder. The SE-SEM micrographs for Al-based nanocomposite powder processed at 360 rpm at 5, 10, 15 and 20 min (**c**–**f**) show the progressive formation of flake-like particles, especially for nanocomposite powder milled at 10, 15 and 20 min, which is the result of a material overmilling.

SEM micrographs for Al powder matrix, MWCNTs and milled Al-based nanocomposite powders are shown in Figure 1. As shown in Figure 1a, the Van der Waals forces [16] between MWCNTs create CNT clusters, which can be deagglomerated during the milling dispersion of the Al-based nanocomposite powder. Figure 1b shows the morphology condition of the as-received Al matrix powder, which exhibits particles with elongated and irregular shapes. The nanocomposite powders were processed using 0.5 wt% of MWCNTs since it was previously demonstrated [2,3] that such a CNT content can help us to achieve an enhancement in the mechanical and electrical properties of sintered nanocomposites. 

We have investigated the CNT dispersion and Al powder morphology by fixing the milling speed at 360 rpm and varying the milling time from 5, 10, 15 and 20 min.

The powder morphologies obtained after varying the milling times are shown in Figure 1c–f. The morphology of powder particles after completing the milling dispersion at 5 min show that the size distribution is homogenized as a result of two processes, size reduction of larger particles and agglomerations of smaller particles to form new ones. The powder morphology evolution for milling times at 10, 15 and 20 min, show the progressive formation of flake-like particles, which results from the powder overmilling. From such an analysis, it is concluded that the most suitable morphology for the sintering of powder nanocomposites is achieved when it is processed at 360 rpm for 5 min.

### 4.2. Morphological Analysis of Industrial Attrition Milling Nanocomposite Powder

Despite the materials used in this research being the same as those used in [3], the milling conditions must be modified, since the amount of material processed is much higher in order to reach conditions to scale up the process to produce raw material with good repeatibility to generate reliable parts using the obtained Aluminum-based nanocomposite material. To attach the CNT to the Al powder particles and to reach a good homogeneous dispersion, the milling speed is tuned to the value of 215 rpm and the time milling process to 5 min. These process parameter values help us to reproduce a similar powder condition as previously reported in [3].

**Figure 2:**
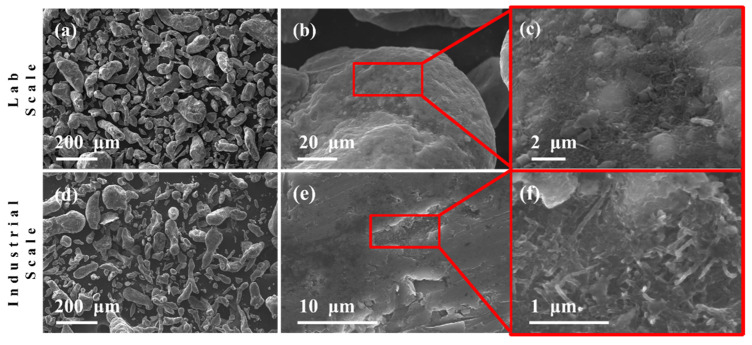
SE-SEM micrographs for Al-MWCNTs nanocomposite powders; (**a**) Laboratory and (**d**) Industrial scale. MWCNTs dispersion within the ball-milled Al powder surface; (**b**,**c**) Laboratory and (**e**,**f**) Industrial scale.

Figure 2 shows the influence of the ball-milling and attrition milling processes in reducing the powders particle size and how the smallest ones were agglomerated to form bigger particles, resulting, in general, in a homogenization in powder morphology, as shown in Figure 2a,d. The zoom-in view of Figure 2b,e confirms good MWCNT dispersion embedded into the Al powder surface by a ball milling process for the preparation of the nanocomposite powder.

In the case of preparation and the nanocomposite powder by attrition milling, several trials were run (not reported here) to find the process parameter values needed to generate similar powder morphologies obtained by the ball-milling method. Figure 2e,f show SEM images confirm that MWCNTs used to reinforce the Al matrix were homogeneously dispersed. Thus, the attrition milling process can be used for the production of Al nanocomposite powders on an industrial scale with similar conditions to those obtained in [3]. 

### 4.3. Sintering Consolidation and Densification at the Lab and Industrial Scale

**Figure 3:**
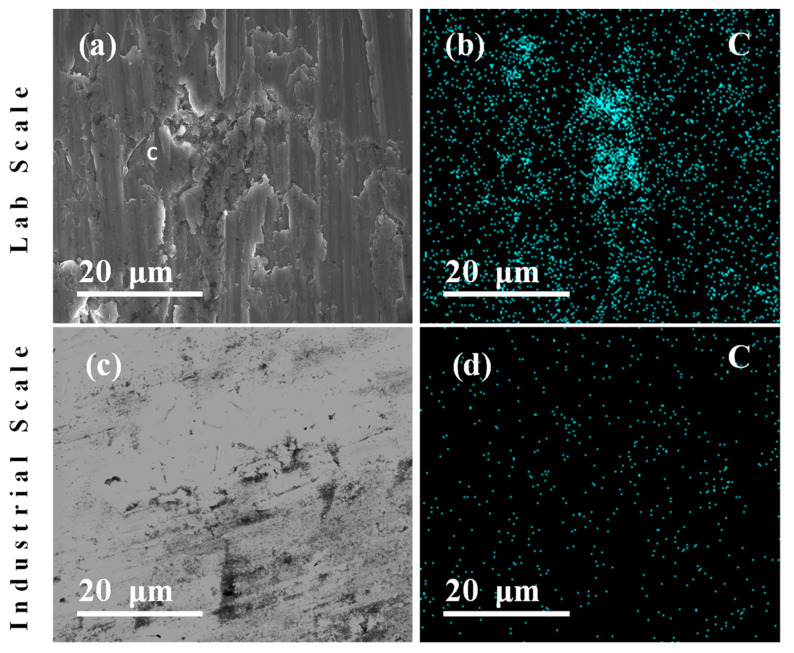
EDS-SEM micrographs for the analysis of carbon dispersion in the obtained sintered nanocomposite specimens. (**a**,**b**) Laboratory process and (**c**,**d**) Illustrates samples made from the industrial process that elucidate homogenous distribution through EDS-SEM elemental mapping carbon (C) analysis.

The analysis of MWCNTs dispersion of the sintered samples is shown in Figure 3. Figure 3a,b illustrate sintered samples resulted from the milling dispersion at 360 rpm for 5 min, confirming that the MWCNTs were successfully dispersed and attached to the Al powder surface at the lab scale. Furthermore, these cross-section SE-SEM images of sintered nanocomposite samples show small fractures. We believe that these fractures could be caused by the preparation of the samples and the elemental mapping analysis for Carbon, corroborating that the MWCNTs are homogenously dispersed within the sintered nanocomposites.

Figure 3c,d shows images of the Al nanocomposite powder obtained using the attrition milling (industrial scale process) when using the machine operation parameter values of 215 rpm by 5 min, and adding PCA that produces an homogeneous distribution of the MWCNT over the Al surface. Figure 3b,d show EDS-SEM elemental mapping that elucidates homogenous distribution through Carbon analysis. 

The obtained results for the nanopowder preparation and sintered materials for both environments, lab scale and scale-up industrial production levels, are similar and thus, one can conclude that the adjusted parameters of such as speed and milling time work well to produce the nanocomposite powder with the addition of the PCA during the milling process at the industrialized level.

The homogenous MWCNTs dispersion and the modified morphology of Al powder particles achieved during the milling process promoted an efficient particle interaction during the sintering process, resulting in a high densification behavior of obtained sintered nanocomposites for both laboratory (samples M1–M10) and industrial processes (samples I1–I2), as summarized in Table 1. In both processes, the low porosity content is attributed to the homogenous MWCNT dispersion. When the MWCNTs are present in large-sized clusters or agglomerations, these act as barriers and hinder the densification process. Such results agree with those reported in [3], where Al-based nanocomposite specimens were produced using the same materials. These results illustrate a behavior similar to that obtained on a lab scale, corroborating that the MWCNTs have been homogeneously dispersed, avoiding the presence of closed pores in the sintered samples.

### 4.4. Surface Finishing Analysis of the Sintered Al-Based Nanocomposites

**Figure 4:**
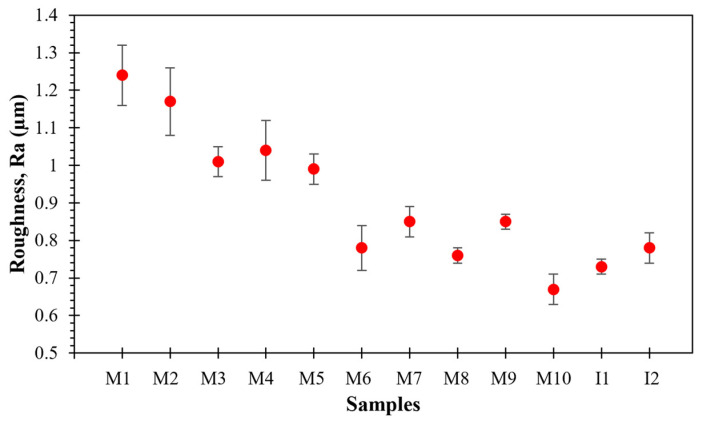
Average roughness (*Ra*) values retrieved from the surface of obtained sintered nanocomposites.

The study of surface-finishing conditions of obtained sintered nanocomposites at both the laboratory scale and at the scale-up industrial production level, was studied via roughness analyses. The average roughness (*Ra*) values shown in Figure 4 were collected from the left sample end, and from the middle and right sample end of the sintered nanocomposites. As a first approach, one can observe that the *Ra* values of the laboratory-sintered nanocomposite surfaces vary from 0.6 to 1.2 μm, which indicates that the surface finishing condition is strongly affected by several phenomena during the sintering of nanocomposite powders like the compaction process, temperature or holding [14]. Additionally, Mackie et al. in [17] reported that the surface roughness can be affected by the use of graphite paper, which can induce a carbon uptake and distribution during the sintering parts by SPS processes. The *Ra* values for the industrial scaled nanocomposites are around of 0.7 μm.

**Figure 5:**
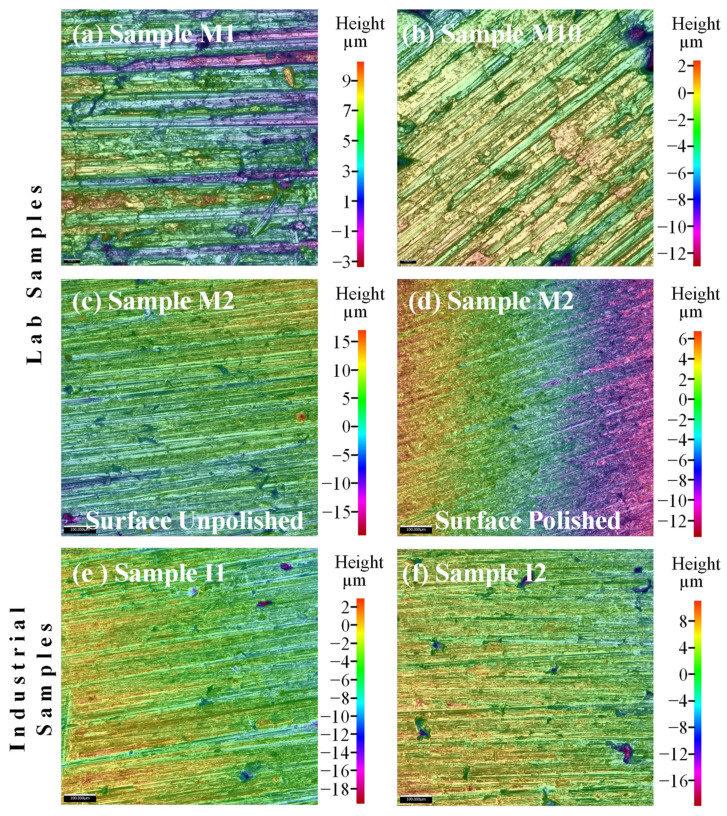
Mapping of the surface roughness for lab samples (**a**) M1, (**b**) M10, as well as for sample M2 with (**c**) surface unpolished, and (**d**) surface polished. Mapping of the surface roughness for samples produced under industrial scale process parameter values (**e**) sample I1, and (**f**) sample I2.

When the lab scale sample surface is finely polished by using silicon carbide sandpapers (600, 800 and 1200 grit sizes), the obtained *Ra* is about 0.4 μm, which, at the same time, is close to the *Ra* value obtained for the surfaces of as-received sintered samples processed at the industrial level. In general, the images taken for the as-received surface reveal the presence of linear patterns (Figure 5) caused by the contact between nanocomposite powders and die during the sintering consolidation. The reconstructed 2D mapping of the surface roughness for samples M1 and M10, corresponding to the roughest and the smallest rough surfaces, is shown in Figure 5a,b. Both figures show that the distance of the maximum peak to valley height is higher, at about 185% for M1, than the distance observed for M10. The latter observation allows us to obtain a more detailed analysis and a better understanding for the formation of roughness in this type of consolidation. Figure 5c,d shows the 2D mappings of surface roughness for sample M2 before and after the polish procedure and contrary to samples M1 and M10, the distance of the maximum peak to valley height in both conditions exhibit a decrease of about 37% after polishing. Similarly, Figure 5e,f show the 2D mapping of the surface roughness for samples I1 and I2 sintered and consolidated in an industrial scale process. 

### 4.5. Micro-Hardness Analysis of Sintered Al-Based Nanocomposites

The results of hardness Vickers tests are summarized in Table 2. The recorded Vickers values in all sintered nanocomposite parts produced at the lab scale are, ranging from 621 to 782 MPa. Such variations could be attributed to the presence of MWCNT clusters arbitrarily located in the boundaries of the Al grains within the sintered samples (as shown in Figure 3b for EDS-SEM Carbon analysis), limiting their growth and resulting in the obtention of different sizes [18]. One can also see from Table 2 that the tensile and yield strength values vary between samples, by about 20 percent. This variation in mechanical strength is consistent with the hardness of experimental measurements. In general, these experimental data confirm the reinforcement achieved during both milling dispersion/adhesion and sintering processes. 

On the other hand, experimental data show that the hardness of sample I1 and I2 increase by approximately 58% with respect to sample M1. The fraction of MWCNT concentrations used in both samples is 0.5% wt with respect to the metallic matrix. Of course, there are different factors that cause hardness to be affected during the consolidation of the nanocomposite samples. For instance, the decrease in hardness is coincident with the significant increase in porosity. Simoes et al. [19] also found that the hardness of the composite is higher than the monolithic aluminium if the CNTs are well dispersed. The hardness of the composite may decrease if the CNTs are damaged during the dispersion process. 

In spite of having hardness values for the nanocomposites, sintered samples at the lab scale are slightly lower than those reported in [2], and the MWCNT dispersion was considerably improved by avoiding the formation of large-sized MWCNT clusters. As previously discussed, having large-sized clusters of MWCNT surrounding the Al grains enhances the hardness sample properties, as confirmed in [2]. As expected, at the industrial scale, the volume of Al-based nanocomposite powder processed is higher than that reported in [2,20,21]. However, the hardness values collected from the nanocomposites sintered and the consolidated samples using the industrial process, are higher than those reported in [2], which is an indication of the scalability of the process.

### 4.6. Electrical Conductivity of Sintered Al-Based Nanocomposites

**Figure 6:**
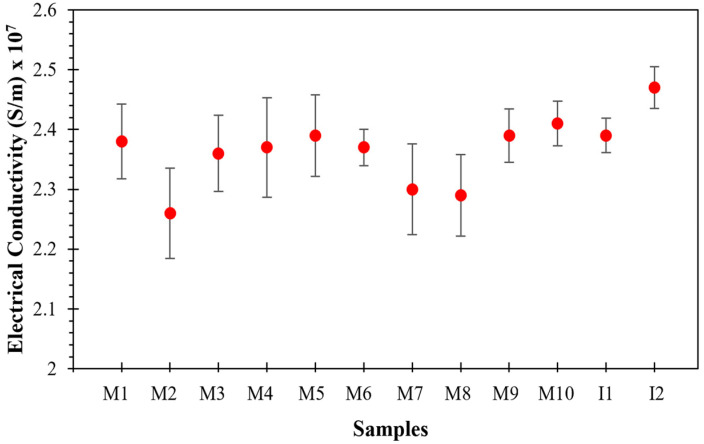
Measured sample electrical conductivity values.

The electrical conductivity values collected from sintered nanocomposite samples are shown in Figure 6 and are summarized in Table 3. In general, the electrical conductivity values are about 40% of the International Annealed Copper Standard (IACS), which agrees with the results and analyses previously discussed. It is well-known that the electron transport in this kind of nanocomposite is mainly promoted by a tunneling effect and percolation phenomena, which at the same time depends on the spatial distribution of the MWCNT reinforcement within the Al matrix, as well as the high densification behavior of sintered nanocomposites. In combination, both provide the necessary conditions to create an effective MWCNT network, which enhances the electron transport within the material. 

The electrical conductivity values collected from the material samples made from the Al-nanocomposite powders reinforced with 0.5 wt% of MWCNTs and produced via industrial processing are similar to those recorded in [2,3]. These findings are of great relevance, since the aim of this research is to explore the scalability of the Al-based nanocomposites that can be used for several engineering applications since both electrical conductivity and mechanical properties tend to increase when using industrial processes to prepare the Al-nanocomposite powders.

## 5. Discussion

Although the experimental conditions were slightly modified for the preparation of samples when scaling the processing of nanocomposite materials in the industrial scale, the obtained sintered nanocomposites show increased mechanical and electrical properties in comparison to those processed and sintered at the lab scale. The latter is mainly attributed to the homogenous dispersion of MWCNTs performed by an attrition milling process, in which the strategy consisted of reproducing the powder morphology previously obtained in [3]. The attrition milling of nanocomposite powders were performed by using a 0.5 wt% of acid stearic as a PCA in order to promote a better powder particle interaction, avoiding overheating and cold welding processes in the obtained milled materials. The irregular morphology obtained in milled nanocomposites was key to promoting a better powder particle interaction during the sintering process, which at the same time was conducted to high densification and low porosity percentage values measured in the sintered nanocomposite materials at the industrial level. Notice that the discrepancy in the values for the hardness, tensile and yield strengths, the electrical conductivity of the SPS sintered samples reported in [2,3] with those listed in Table 2 and Table 3 are due to the milling time, the addition of the stearic acid as PCA, the attrition milling conditions, the rotation speed, the holding time, the load for the batch development, and the sintering conditions of the controlled atmosphere sinter hardening furnace equipment.

On the other hand, the surface finishing condition validated through the values and 2D mapping of the surface roughness, show that the nanocomposites produced for industrial mass production is quite a bit better than for those processed at the lab scale. As a result, the mechanical and electrical properties showed an enhancement in their performances, which suggests that the MWCNTs located at the surroundings of the Al grains, as well as their homogenous dispersion, were of relevance to create an effective reinforcement in the metal matrix [2,3]. The data collected for the samples M1 to M10 listed in Table 3 differ from those values collected for samples I1 and I2. We believe that these variations in physical and electrical properties could be due to the addition of the PCA during the milling process of the Al-based nanocomposite powders to be used for large-scale production. In addition, the 12 inch atmosphere sinter hardening furnace equipment was a key factor to gain better process control in combination with the tool used for fabricating parts in a mass production setting environment. In fact, we have identified that during the production of the sintered parts using the controlled atmosphere sinter hardening furnace equipment, the addition of the stearic acid as PCA expedites the powder milling stage. Furthermore, we have noticed that PCA acts as a lubricant during the press stage, which benefits the final part cost, since the surface roughness values are in accordance with those needed for mass production. In summary, the controlled atmosphere sinter hardening furnace equipment with the appropriate process parameter is a valued and key factor in the manufacturing of large batches of functional parts without any further post-machining part process.

Finally, we would like to mention that during the process to scale-up the developed material, some Siemens partner and suppliers were reluctant to invest money in transforming their production line processes for the part fabrication because of the unknown effects that could occur during production. Siemens partners and suppliers were also concerned about the time needed to adapt their production technologies to the new nanostructure metallic powders. They were also concerned about the negative effects that the machine tooling could experience during the production process, since their replacement or repair cost might increase the market price of each functional piece. Another concern that comes into play is linked to the contamination of the production line due to the usage of the developed nanostructured metallic powders, as well as the addition of the process control agent (PCA). Furthermore, Siemens and the research-working group from Tec de Monterrey explored other manufacturing techniques, such as the advanced stir-casting process, which is suitable for the mass production of composite metallic powders. An overview of the advantages and disadvantages of this technology when compared with other fabrication technologies is provided by Musa et al. in [22]. However, this technology was discarded because of its limitations to produce the intricate part shapes needed by Siemens. We also found that in this process, metallic powders, such as aluminum, can spontaneously burn and high compaction forces are needed. Therefore, continuous machine inspections will be required to ensure that high quality production and high load capacity machines will be produced for the mass production process influencing machine energy consumption and the part final price.

## 6. Conclusions

In this paper, a controlled atmosphere sinter hardening furnace equipment was used to investigate Al-based nanocomposites for industrial mass production in order to find the best experimental conditions capable of preserving or enhancing their mechanical and electrical properties. The strategy implemented focuses on reproducing the first experimental results obtained in the lab scale environment and then, by translating the process to the industrial scale and ensuring the desirable mechanical and physical properties to produce parts that fulfill with industry requirements and regulations. The main outcomes of this study can be summarized as follows:

- The CNT distribution in the Al matrix at the industrial scale was performed by substituting the ball-milling equipment for an attrition milling process, which is ideal for the processing of high material volume. To attain the desired morphology of the milled nanocomposite powders for industrial mass production, we adjust the processing parameters and use stearic acid as a PCA.
- During the mass production of the nanocomposite powders, their morphology was successfully controlled by promoting a homogenous distribution of the MWCNT, which is similar to that obtained at the lab scale.
- The equipment used for the consolidation and densification of the obtained specimens for industrial mass production showed high densification values (around 99.2%), which are quite similar to those recorded in lab-made samples.
- During the surface finishing of the produced parts, the industrial-scaled specimens and surface roughness values are found to be lower than those measured for the samples produced following lab setting conditions after surface treatment (polishing). This is mainly due to the tool used, which has an electropolished surface that allows us to produce functional parts without any further post-process or machining operation.
- The homogenous CNT distribution during the milling of nanocomposite powders in combination with their corresponding sintering consolidation process promotes an effective reinforcement in the sintered samples for the industrial scale, which influences the hardness, tensile and yield strength values, which were around 58% higher than those values measured for the produced lab samples.
- The electrical conductivity values recorded from the mass production of metallic powder samples are about 42%IACS, which are consistent with those recorded from the produced lab-scale samples.

In summary, this study unveils the steps that one could follow to scale from the lab to mass production metallic parts produced via Al powders reinforced with MWCNTs with enhanced mechanical and electrical properties.

## Figures and Tables

**Table 1:** Relative density and porosity percentage calculated for ten sintered Al-based nanocomposite samples. M and I labels are used for the samples processed and sintered at the laboratory and industrial level, respectively.

Sample	Relative Density (%)	Porosity (%)	Sample	Relative Density (%)	Porosity (%)
M1	98.2	1.80	M6	98.2	1.80
M2	99.7	0.30	M7	98.6	1.40
M3	98.8	1.20	M8	98.6	1.40
M4	99.4	0.60	M9	99.2	0.80
M5	98.2	1.80	M10	99.0	1.00
I1	99.2	0.80	I2	99.1	0.90

**Table 2:** Micro-hardness, tensile and yield strength values of SPS Al-based nanocomposites.

Sample	Hardness Vickers (MPa)	Tensile Strength (MPa)	Yield Strength (MPa)
M1	692.3 ± 21.3	234.8 ± 7.25	145.6 ± 4.50
M2	700.2 ± 46.1	237.5 ± 15.6	147.2 ± 9.70
M3	713.9 ± 18.1	242 ± 6.17	150.1 ± 3.82
M4	629.6 ± 14.3	213.5 ± 4.86	132.4 ± 3.01
M5	782.6 ± 11.7	265.5 ± 4.00	164.6 ± 2.47
M6	678.6 ± 31.6	230.2 ± 10.7	142.7 ± 6.66
M7	641.3 ± 15.7	217.5 ± 5.32	134.9 ± 3.30
M8	649.2 ± 32.1	220.2 ± 10.9	136.5 ± 6.75
M9	621.7 ± 22.2	210.9 ± 7.55	130.7 ± 4.68
M10	674.7 ± 19.2	228.9 ± 6.51	141.9 ± 4.04
I1	957.1 ± 94.7	324.7 ± 32.1	201.3 ± 19.9
I2	1190.5 ± 139.6	403.8 ± 47.3	250.4 ± 29.3

**Table 3:** Micro-hardness, tensile and yield strength values of SPS Al-based nanocomposites.

Sample	Conductivity (S/m)	%IACS	Sample	Conductivity (S/m)	%IACS
M1	$2.32\;\times \;10^{7}$	40.11	M6	$2.38\;\times \;10^{7}$	41.02
M2	$2.26\;\times \;10^{7}$	39.10	M7	$2.30\;\times \;10^{7}$	39.80
M3	$2.36\;\times \;10^{7}$	40.78	M8	$2.29\;\times \;10^{7}$	39.59
M4	$2.37\;\times \;10^{7}$	40.90	M9	$2.39\;\times \;10^{7}$	41.21
M5	$2.39\;\times \;10^{7}$	41.22	M10	$2.41\;\times \;10^{7}$	41.60
I1	$2.39\;\times \;10^{7}$	41.36	I2	$2.47\;\times \;10^{7}$	42.57

## Data Availability

The data is available on reasonable request from the corresponding author.
